# Lasting immune memory against hepatitis B in children after primary immunization with 4 doses of DTPa-HBV-IPV/Hib in the first and 2^nd ^year of life

**DOI:** 10.1186/1471-2334-10-9

**Published:** 2010-01-15

**Authors:** Michael Steiner, Gunasekaran Ramakrishnan, Britta Gartner, Olivier Van Der Meeren, Jeanne-Marie Jacquet, Volker Schuster

**Affiliations:** 1 Medical Practice, Werderstr. 3, 88348 Bad Saulgau, Germany; 2 Clinical Statistics, GlaxoSmithKline Biologicals, Bangalore, India; 3 Med. Fachbereich Impfstoffe, GlaxoSmithKline Biologicals, Munich, Germany; 4 Clinical Department, GlaxoSmithKline Biologicals, Rixensart, Belgium; 5 University Hospital for Children and Adolescents, Leipzig, Germany

## Abstract

**Background:**

Few studies have assessed long term persisting immunity against hepatitis B virus (HBV) in children vaccinated during infancy with combined vaccines containing recombinant HBV surface antigen (HBs). We assessed antibody persistence and immune memory in children 4-5 years of age, previously vaccinated with four doses of combined hexavalent DTPa-HBV-IPV/Hib vaccine (*Infanrix hexa*™).

**Methods:**

Immune memory was assessed in 301 children through administration of a challenge dose of monovalent HBV vaccine.

**Results:**

At 4-5 years of age, 85.3% of subjects had persisting anti-HBs antibody concentrations ≥ 10 mIU/mL, rising to 98.6% after the HBV challenge dose. All but 12 subjects (95.8%) achieved post-challenge anti-HBs concentrations ≥ 100 mIU/mL. The post-challenge anti-HBs GMC rose by 100-fold compared to pre-challenge concentrations. An anamnestic response to the HBV vaccine challenge was observed in 96.8% of subjects, including 17/21 (81.0%) of children with initially undetectable antibodies (<3.3 mIU/mL). All but 4 of 42 subjects (90.5%) with anti-HBs antibodies <10 mIU/mL prior to the challenge dose, achieved seroprotective levels afterwards. A 4-fold rise in antibody concentration after the challenge dose was observed in 259/264 (98.1%) of initially seropositive subjects. The magnitude of the post-challenge responses was proportional to pre-challenge anti-HBs levels. No serious adverse events were reported during the study.

**Conclusion:**

The combined DTPa-HBV-IPV/Hib vaccine induced lasting immune memory against hepatitis B. Long term protection afforded by DTPa-HBV-IPV/Hib is likely to be similar to that observed following priming with monovalent HBV vaccines.

**Trial registration:**

http://www.clinicaltrials.gov 106789 NCT00411697

## Background

Achieving high routine vaccination coverage against hepatitis B in infancy is considered the highest priority for hepatitis B prevention by the World Health Organization (WHO) [1]. Universal Infant vaccination as the primary prevention strategy was adopted by the WHO in 1988 [2], after the failure of vaccination strategies targeting only at-risk groups [3,4]. Infant vaccination has the greatest impact on preventing chronic hepatitis B and its subsequent complications [1]. Furthermore, maintaining high vaccine coverage is more sustainable in infants than in adolescents who are difficult to reach and frequently poorly compliant [3,5-7].

Combination vaccines for use in infancy have an increasingly important role in contributing to high levels of parental acceptance of vaccination. Combination vaccines reduce the number of injections required for full vaccination and improve the timeliness of vaccination, thereby contributing to maintaining high levels of vaccine coverage [8,9]. Several combined vaccines containing hepatitis B vaccine are currently commercially available, the largest of which is the hexavalent diphtheria-tetanus-pertussis-hepatitis B-inactivated poliomyelitis and *Haemophilus influenzae *type b conjugate vaccine (DTPa-HBV-IPV/Hib) manufactured by GlaxoSmithKline Biologicals (GSK, Rixensart, Belgium).

DTPa-HBV-IPV/Hib is licensed for primary vaccination of infants and for second year of life booster vaccination in many countries throughout the world, including all European Union countries. Previous clinical studies have shown DTPa-HBV-IPV/Hib to be well tolerated and immunogenic [10]. In particular, three dose primary vaccination with DTPa-HBV-IPV/Hib induces seroprotective antibody levels (anti-HBs ≥ 10 mIU/mL) against hepatitis B in over 95% of subjects [10], comparable to results following monovalent hepatitis B vaccines [10,11]. This study expands upon these previous reports of DTPa-HBV-IPV/Hib by assessing the persistence of immunological memory in children between 4 and 5 years of age who had been previously primed and boosted with four doses of DTPa-HBV-IPV/Hib in their first two years of life.

## Methods

The study was an open-label serological follow up study (http://www.clinicaltrials.gov 106789 NCT00411697) conducted in 27 centers in Germany, between 19 December 2006 and 14 May 2007. The study was conducted according to Good Clinical Practice guidelines, the Declaration of Helsinki, and applicable German laws. The study protocol was approved by Ethik-Kommission der Landesärztekammer Baden-Württemberg, Jahnstraße 40, 70597 Stuttgart. Written informed consent was obtained from parents/guardians before enrolment.

All subjects were healthy and previously vaccinated with four doses of DTPa-HBV-IPV/Hib (*Infanrix hexa*™; GSK Biologicals) administered via routine immunization procedures in Germany. The recommended infant vaccination schedule in Germany is at 2, 3 and 4 months of age. Since strict adherence to the schedule could not be guaranteed, subjects were to have received three primary vaccination doses by 9 months of age and one booster dose received between 11 and 18 months of age. Subjects who had received hepatitis B vaccination at birth, or any previous hepatitis B booster vaccination after administration of the fourth DTPa-HBV-IPV/Hib dose in the second year of life were excluded.

Enrolled children received a single challenge dose of monovalent pediatric hepatitis B vaccine (*Engerix*™-B Kinder, containing 10 μg recombinant hepatitis B surface antigen [HBs], GSK) administered intramuscularly into the deltoid. A blood sample was collected before and one month after the hepatitis B challenge dose. Anti-HBs antibodies were measured using GSK Biologicals' in-house ELISA with a cut-off of 3.3 mIU/mL defining seropositivity [12]. Anti-HBs antibody concentrations ≥ 10 mIU/mL were considered to be seroprotective.

Serious adverse events occurring within 30 days of vaccination were recorded.

The primary analysis of immunogenicity was conducted on the according-to-protocol (ATP) immunogenicity cohort comprising subjects who complied with protocol defined procedures and criteria, who received the challenge hepatitis B vaccine dose, and for whom immunogenicity data were available.

The percentage of subjects with anti-HBs antibody concentrations ≥ 10 mIU/mL and ≥ 100 mIU/mL, with exact 95% confidence intervals (CI), prior to and one month after the challenge dose, was calculated. Persistence of immunological memory shown by the ability to mount an anamnestic response to a challenge dose of hepatitis B vaccine, was defined as anti-HBs concentrations ≥ 10 mIU/mL in subjects seronegative (i.e. anti-HBs concentrations <3.3 mIU/mL) before the challenge and as an increase of at least 4-fold in anti-HBs concentrations in subjects seropositive (anti-HBs concentrations ≥ 3.3 mIU/mL) before the challenge.

## Results

In total, 301 subjects were enrolled and vaccinated. The ATP immunogenicity cohort consisted of 286 subjects. Subjects were eliminated from the ATP immunogenicity analysis because they had received forbidden vaccines; had not received the full DTPa-HBV-IPV/Hib course in infancy; had not complied with the blood sampling schedule; had missing serological data; had blood samples that could not be reliably identified; or because they had tested positive to anti-hepatitis B core antigen (one subject, 0.3%).

The mean age of subjects in the ATP immunogenicity cohort was 4.5 years (standard deviation [SD] 0.5), of which 51.7% (148) were female. The majority of subjects were Caucasian.

Prior to the booster dose, 85.3% (250/293) of DTPa-HBV-IPV/Hib primed and boosted subjects had persisting anti-HBs levels ≥ 10 mIU/mL. This rose to 98.6% (282/286) after the HBV challenge dose (Table 1) and the post-challenge anti-HBs antibody GMC rose by 100-fold compared to pre-challenge concentrations. All but 12 subjects (95.8%) achieved post-challenge anti-HBs antibody concentrations ≥ 100 mIU/mL.

**Table 1:** Anti-HBs antibody responses before and one month after challenge dose (ATP Cohort for immunogenicity)

		Seropositivity/Seroprotection rates										
		≥ 3.3 mIU/mL			≥ 10 mIU/mL			≥ 100 mIU/mL			GMC	
Time point	N	n	%	[95% CI]	n	%	[95% CI]	n	%	[95% CI]	Value	[95% CI]
Pre	285	264	92.6	[89.0; 95.4]	243	85.3	[80.6; 89.2]	134	47.0	[41.1; 53.0]	83.7	[66.8; 104.9]
Post	286	284	99.3	[97.5; 99.9]	282	98.6	[96.5; 99.6]	274	95.8	[92.8; 97.8]	8656.1	[6560.7; 11420.9]

GMC = geometric mean antibody concentration. N = number of subjects with available results. n/% = number/percentage of subjects with antibody concentrations above the specified cut-off. 95% CI = exact 95% confidence interval. Pre = prior to the administration of challenge dose, Post = one month after the challenge dose

**Figure 1:**
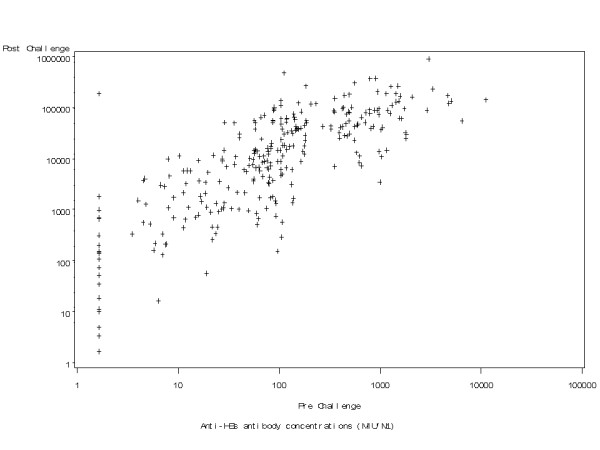
**Relationship between anti-HBs antibody concentrations before and after HBV challenge (ATP Immunogenicity cohort)**.

An anamnestic response to the hepatitis B vaccine challenge was observed in 96.8% [95% CI 94.1; 98.5] of subjects (276/285). Of 21 subjects whose pre-challenge anti-HBs antibody level was below 3.3 mIU/mL (i.e. seronegative), 17 (81.0%) reached the 10 mIU/mL seroprotection cut off after the challenge dose. Of 42 subjects who had anti-HBs antibody concentrations below 10 mIU/mL prior to the challenge dose, all but 4 (90.5%) achieved seroprotective anti-HBs levels after the challenge (Table 2). Of 264 subjects with detectable anti-HBs antibodies prior to the challenge dose, 98.1% (259) had at least a 4-fold increase in antibody concentrations following the challenge dose. The magnitude of the post-challenge responses was proportional to pre-challenge anti-HBs levels (Table 2, Figure 1).

**Table 2:** Anti-HBs antibody responses one month after HBV challenge by pre-vaccination status (ATP Cohort for immunogenicity)

					Post challenge							
		≥ **3.3 mIU/ml**			≥ **10 mIU/ml**			≥ **100 mIU/ml**			GMC	
Pre-challenge status	N	n	%	95% CI	n	%	95% CI	n	%	95% CI	value	95% CI
<3.3 mIU/ml	21	19	90.5	[69.6; 98.8]	17	81.0	[58.1; 94.6]	11	52.4	[29.8; 74.3]	85.4	[24.7; 295.0]
≥ 3.3 mIU/ml	264	264	100	[98.6; 100]	264	100	[98.6; 100]	262	99.2	[97.3; 99.9]	12524.1	[9907.2; 15832.2]
<10 mIU/ml	42	40	95.2	[83.8; 99.4]	38	90.5	[77.4; 97.3]	31	73.8	[58.0; 86.1]	249.8	[116.9; 534.1]
≥ 10 mIU/ml	243	243	100	[98.5; 100]	243	100	[98.5; 100]	242	99.6	[97.7; 100]	16010.1	[12812.2; 20006.2]
<100 mIU/ml	151	149	98.7	[95.3; 99.8]	147	97.4	[93.4; 99.3]	139	92.1	[86.5; 95.8]	2119.3	[1475.8; 3043.4]
≥ 100 mIU/ml	134	134	100	[97.3; 100]	134	100	[97.3; 100]	134	100	[97.3; 100]	42433.2	[34084.1; 52827.5]

N = number of subjects with available results. n/% = number/percentage of subjects with antibody concentrations above the specified cut-off. 95% CI = exact 95% confidence interval.

No serious adverse events were reported during the study.

## Discussion

Primary and booster vaccination in the first two years of life with DTPa-HBV-IPV/Hib induced long lived immunological memory against hepatitis B in subjects between 4 and 5 years of age who had been primed and boosted via routine pediatric services in Germany. These data are remarkably similar to data obtained from cohorts of subjects 4 to 6 years of age primed with DTPa-HBV-IPV/Hib in clinical trial settings in Germany, where adherence to the primary and booster vaccination schedule was likely to have been more strictly enforced [13,14]. In these studies, anti-HBs antibody levels associated with seroprotection were observed between 86.0% and 91% of DTPa-HBV-IPV/Hib trial-vaccinated subjects at 4-6 years of age [13,14], and an anamnestic response following HBV vaccine challenge was observed in 95.7% [14]. A recent follow-up study conducted in Italy in children in the third year of life, primed with DTPa-HBV-IPV/Hib at 2, 3 and 11/12 months of age, also showed high persisting seroprotective anti-HBs levels (96.0% of subjects) and anamnestic responses to a challenge HBV vaccine dose in those with concentrations <10 mIU/mL [15]. Together, these results provide strong evidence for the immunogenicity of the hepatitis B component of DTPa-HBV-IPV/Hib in routine use.

Booster doses of hepatitis B vaccine after primary vaccination in immunocompetent individuals are currently not recommended by either the WHO [16] or the European Consensus group on Hepatitis B Immunity [17]. Decrease of antibody concentrations below seroprotection level or even below detection levels is not considered as an indicator of loss of protection. Persisting protection against clinical hepatitis B disease and chronic hepatitis B carriage has been shown to last for up to two decades [17-20].

A potential limitation of the study is that the analysis was performed, as pre-planned in the protocol, on ATP immunogenicity cohort. However, fewer than 5% of subjects (15/301) were eliminated from the ATP cohort, and of these, blood samples were not available or could not be reliably identified for 5 subjects. It is therefore unlikely that the conclusions from an analysis of the Total cohort, with data from only 10 additional subjects, would differ from the ATP analysis. The findings of the present study are important because they confirm the medium term immunogenicity of hepatitis B vaccination by using a hexavalent combination vaccine in subjects from a low endemicity region who are unlikely to have been exposed to hepatitis B, and who were primed with a large combination vaccine. Further serological follow-up studies of older individuals until age 15 years, who received four consecutive doses of DTPa-HBV-IPV/Hib as primary and booster vaccination in routine vaccination practice are planned, and will be performed as vaccinated cohorts become older.

## Conclusions

In conclusion, primary and booster vaccination with DTPa-HBV-IPV/Hib in infancy induces sustained seroprotection against hepatitis B up to the age of 4-6 years. A strong response to hepatitis B vaccine challenge at this age was demonstrated, indicative of persisting immune memory, even in subjects who had lost circulating antibodies. Longer follow-up is required to ascertain the presence of immune memory at later ages.

## Competing interests

This study was funded by GlaxoSmithKline Biologicals. Drs B Gartner, R Gunasekaran, JM Jacquet and O Van Der Meeren are employees of GlaxoSmithKline Biologicals. VS received investigator fees from GSK.

All authors participated in the design or implementation, analysis and interpretation of the study, the writing of the report and the decision to submit the manuscript for publication.

## Authors' contributions

GR participated in the design of the study and performed the statistical analysis. MS, VS, BG, OVM, J-MJ conceived of the study, and participated in its design and coordination and helped to draft the manuscript. All authors read and approved the final manuscript.

## Pre-publication history

The pre-publication history for this paper can be accessed here:

http://www.biomedcentral.com/1471-2334/10/9/prepub
